# Risk Factors for Severe Neonatal Hyperbilirubinemia in Low and Middle-Income Countries: A Systematic Review and Meta-Analysis

**DOI:** 10.1371/journal.pone.0117229

**Published:** 2015-02-12

**Authors:** Bolajoko O. Olusanya, Folasade B. Osibanjo, Tina M. Slusher

**Affiliations:** 1 Director, Centre for Healthy Start Initiative, Ikoyi, Lagos, Nigeria; 2 Maternal and Child Health Unit, Centre for Healthy Start Initiative, Ikoyi, Lagos, Nigeria; 3 Department of Pediatrics, University of Minnesota, Minneapolis, Minnesota, United States of America; 4 Hennepin County Medical Center, Minneapolis, Minnesota, United States of America; University of Alabama at Birmingham, UNITED STATES

## Abstract

**Background:**

Available evidence suggests that low- and middle-income countries (LMICs) bear the greatest burden of severe neonatal hyperbilirubinemia characterized by disproportionately high rates of morbidity, mortality and neurodevelopmental disorders compared to high-income countries. We set out to identify the risk factors that contribute to the burden of severe hyperbilirubinemia in the most developmentally disadvantaged LMICs to highlight areas for action and further research.

**Methods:**

We systematically searched PubMed, Scopus, Ovid EMBASE, Cumulative Index to Nursing and Allied Health Literature (CINAHL), WHO Library Database (WHOLIS), African Index Medicus (AIM), African Journals Online (AJOL), LILACS, and IndMed for reports published between January 1990 and June 2014. We included only studies that controlled for the effects of confounding variables in determining maternal and infant risk factors for severe hyperbilirubinemia. We conducted meta-analysis of the eligible studies and computed the summary risk estimates with random effects models.

**Results:**

A total of 13 studies with 1,951 subjects and 32,208 controls from India, Nigeria, Pakistan, Nepal and Egypt were identified and analyzed. The pooled data showed that primiparity (OR, 1.59; 95% CI:1.26-2.00), delivery outside public hospitals (OR, 6.42; 95% CI:1.76-23.36), ABO incompatibility (OR, 4.01; 95% CI:2.44-6.61), Rhesus hemolytic disease (OR, 20.63; 95% CI:3.95-107.65), G6PD deficiency (OR, 8.01; 95% CI:2.09-30.69), UGT1A1 polymorphisms (OR, 4.92; 95% CI:1.30-18.62), low gestational age (OR, 1.71; 95% CI:1.40-2.11), underweight/weight loss (OR, 6.26; 95% CI:1.23-31.86), sepsis (OR, 9.15; 95% CI:2.78-30.10) and high transcutaneous/total serum bilirubin levels (OR, 1.46; 95% CI:1.10-1.92) placed infants at increased risk of severe hyperbilirubinemia or bilirubin induced neurologic dysfunctions. Low social class was not associated with an increased risk of severe hyperbilirubinemia.

**Conclusions:**

Infants at risk of severe hyperbilirubinemia in LMICs are associated with maternal and neonatal factors that can be effectively addressed by available interventions to curtail the disease burden prevailing in the affected countries.

## Introduction

Some degree of neonatal jaundice or hyperbilirubinemia is an unpreventable condition in 60%–80% of newborns worldwide [1,2]. In a proportion of infants, jaundice may become severe, progressing to acute bilirubin encephalopathy (ABE) or kernicterus with a significant risk of neonatal mortality [3,4]. Surviving infants may acquire long-term neurodevelopmental sequelae such as cerebral palsy, sensorineural hearing loss, intellectual difficulties or gross developmental delays [5,6]. Available clinical guidelines recommend early detection of infants at risk of severe hyperbilirubinemia to facilitate timely and effective prevention of the associated burden [7,8]. Current evidence however, suggests that low- and middle-income countries (LMICs) disproportionately bear the burden of severe neonatal hyperbilirubinemia [9,10]. For example, in one recent review on the global burden of hyperbilirubinemia, sub-Saharan Africa and South Asia were reported as the leading contributors to an estimated 1.1million babies who would develop severe hyperbilirubinemia worldwide every year [9]. Another systematic review found that LMICs consistently report substantially higher rates of exchange transfusion and bilirubin-induced neurologic dysfunctions (acute bilirubin encephalopathy (ABE) and chronic bilirubin encephalopathy or kernicterus) than in high-income countries [10]. The challenge of managing infants with ABE and kernicterus and their sequelae is daunting especially in resource-constrained settings [11–14]. Early identification of infants at risk of severe hyperbilirubinemia is therefore, even more crucial to curtailing the burden of this ubiquitous and potentially devastating condition within the first 14 days of life [15]. However, the underlying risk factors in LMICs have not been systematically explored to guide necessary clinical and public health interventions. This systematic review and meta-analysis therefore, set out to determine the risk factors for severe hyperbilirubinemia in LMICs in line with PRISMA guidelines [16] to inform health care policy and practice in and for the region.

## Methods

### Eligible focus countries and search strategy

The term “LMICs” based on the World Bank classification broadly covers approximately 140 countries with per capita Gross National Income (GNI) ranging from US$150—US$12,615 [17]. In view of this wide gap in income distribution and in order to focus on the most disadvantageous LMICs, we selected the 91 countries with per capita GNI of ≤US$6,000 using the Human Development Report (HDR) 2013 published by United Nations Development Programme (UNDP) as shown in S1 Table [15].

We searched major electronic databases including PubMed, Scopus, Ovid EMBASE and Cumulative Index to Nursing and Allied Health Literature (CINAHL), using the terms “neonatal jaundice” OR “neonatal hyperbilirubinemia” OR “bilirubin encephalopathy” OR “kernicterus” AND “risk factor” for original articles published between January 1990 and June 2014. In WHO Library Database (WHOLIS), Latin American and Caribbean Health Sciences Literature (LILAC), Indian Medical Journals (IndMed), African Index Medicus (AIM) and African Journals Online (AJOL) we simply used the term “jaundice” OR “hyperbilirubinemia” to maximize the search hits. We reviewed the reference lists of retrieved articles as well as relevant systematic reviews. No limits were used to ensure maximum yield of relevant reports.

### Data extraction

Because of the lack of uniform bilirubin thresholds for severe hyperbilirubinemia in the literature and across populations or routine clinical diagnosis based on total serum/plasma bilirubin (TSB) levels, we chose to consider neonatal hyperbilirubinemia requiring immediate treatment with phototherapy and/or exchange transfusion (typically from total serum bilirubin >20mg/dL or 340μmol/L) and occurring within the first 14 days of life as “clinically significant” or “severe”. Our eligibility criteria for studies on risk factors from eligible countries included cohort, cross-sectional or case-control studies with well-defined control groups and statistical adjustment for confounders. Studies with ABE or kernicterus as primary outcomes were also included.

We screened all titles and abstracts based on these criteria to identify studies for inclusion. We excluded all case reports, case series studies, multiple publications on the single cohort from the same author(s), letters to the editor and animal studies. We also excluded studies of subpopulations of infants with specific risk profile such as diabetic mothers, preterm/low birth weight, sepsis, or hemolytic conditions including glucose-6-phosphate dehydrogenase (G6PD) deficiency, maternal-fetal ABO blood group incompatibility and Rh hemolytic disease for failing to adjust for potential confounders. Studies exploring the association between neonatal hyperbilirubinemia and adverse neonatal outcomes such as mortality and neurodevelopmental disorders were excluded. Discrepancies were resolved by discussion in relation to the study objectives. Data extracted from each retrieved article included: name of the first author, year of publication, country where the study was performed, study design, cases/control, sample size, primary outcome(s), diagnostic criterion and risk factors reported. Disagreements between authors were resolved through consensus after joint reassessment.

### Assessment of methodological quality

The methodological quality of included articles was reviewed and cross-checked independently by two authors (FBO and BOO). Due to the lack of standardized quality scoring system for observational studies relevant for our purpose, we chose to examine six important components of quality/risk of bias assessment: selection of subjects (representativeness), sample size, clear eligibility criterion (ascertainment of exposure), and diagnostic criterion for hyperbilirubinemia, primary outcome measurement and control for confounding factors (see S2 Table). This was adopted from a validated protocol for evaluating observational and non-randomized studies [18]. Each component was rated as satisfactory (1) or unsatisfactory (0), especially when the required evidence was unclear or lacking. Overall scores were classified as follows: low risk (good quality) for 5 or 6, medium risk (satisfactory quality) for 3 or 4, or high risk (poor quality) for 0, 1 or 2. Disagreements were resolved by consensus after reassessment or by adjudication of the third author (TMS).

### Data analysis

We examined the risk factors for severe neonatal hyperbilirubinemia (inclusive of ABE and kernicterus). We did not carry out separate analysis for ABE and kernicterus because of the limited number of studies with relevant data and the high but poorly documented risk of these factors among infants with severe hyperbilirubinemia from LMICs [15]. Statistical analyses were performed using the Comprehensive Meta-analysis software (Version 2.0.064, BIOSTAT, Englewood, NJ) [19]. To quantify the risk factors of severe neonatal hyperbilirubinemia, we calculated pooled odds ratios (ORs) and 95% confidence intervals (CIs) by using the DerSimonian-Laird random-effects models, and Z-statistic test for overall effect was done. The random rather than fixed-effects model was chosen as we anticipated a priori that eligible studies would be from different populations and have three different designs: cohort, cross-sectional and case-control. P<0.05 was considered to be statistically significant. The statistical heterogeneity among each study was assessed by using the Cochran’s Q and I2-statistic tests in order to gain better insights on the degree of heterogeneity in the included studies. Heterogeneity based on the Q statistic was considered significant when p<0.1. I2 values of <25% and >50% reflects low and high heterogeneity, respectively [20]. Publication bias in pooled data involving three or more studies was assessed using Egger’s test. Sensitivity analysis was performed to examine the effect of removing one study in each turn on the outcomes. Dose-response analysis was not performed because of the limited number eligible studies.

## Results

### Study selection

**Fig 1:**
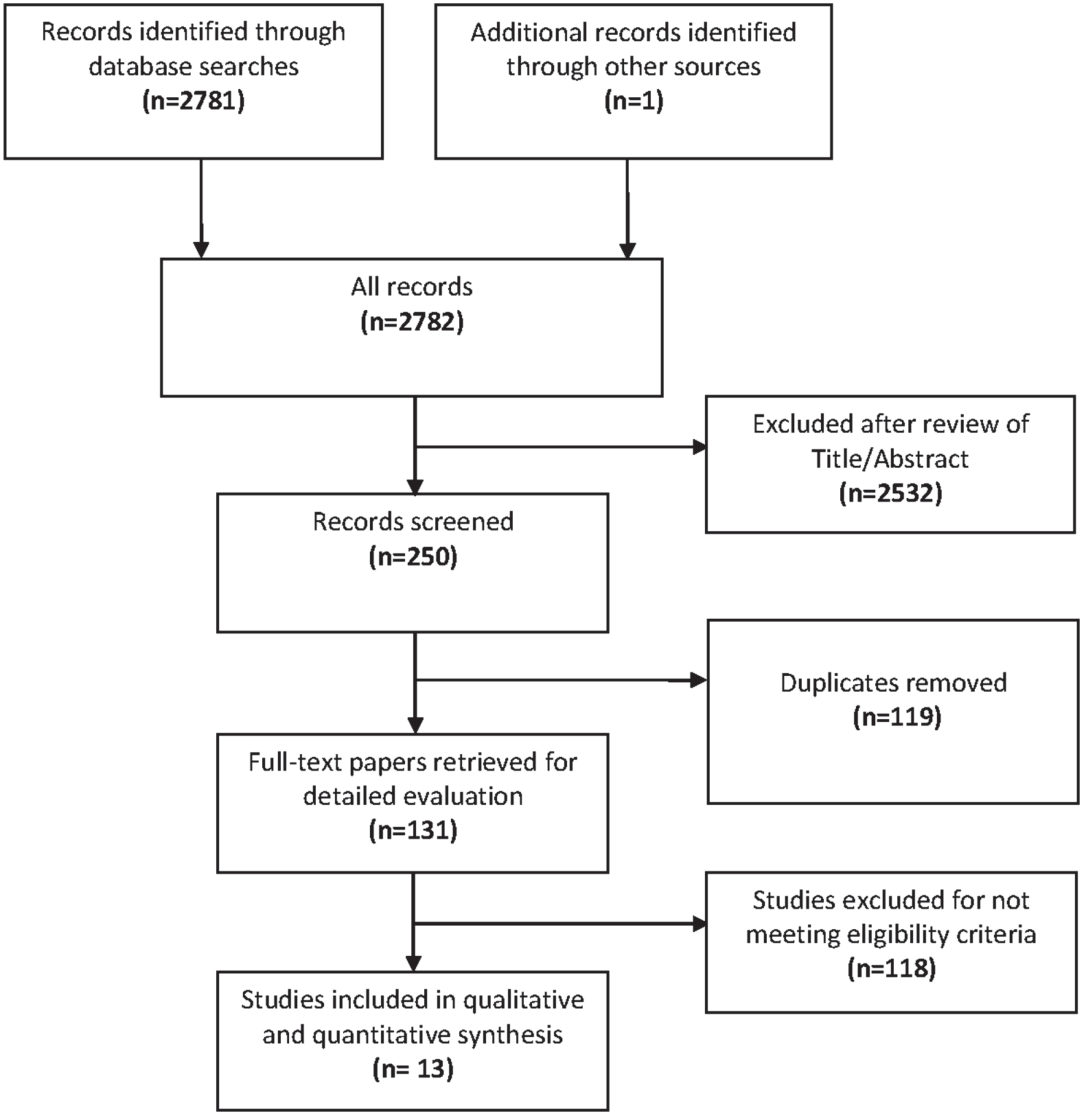
Flow diagram of the study selection process and results.

The initial search across all databases yielded 2,781 studies and one additional study was retrieved from a review paper (Fig. 1). After assessment of titles and abstracts, 250 studies were assembled from all sources, out of which full-texts for 131 studies were required after excluding duplicates. A total of 13 studies with 1,951 cases and 32,208 controls met our eligibility criteria and were selected for final analysis [21–33]. Prior to excluding duplicates, PubMed (n = 82) had the highest hits for LMICs, followed by Scopus (n = 69), Embasse (n = 59), LILAC (n = 12), AJOL (n = 5) and IndMed (1). No record was retrieved from WHOLIS (data not shown).

### Characteristics of included studies

The characteristics of the 13 studies included in this review are summarized in Table 1. Six of the studies were cross-sectional, five were cohort studies and two were case-control studies, all published between 1995 and 2014. Five studies were conducted in India, four in Nigeria, two in Nepal and one each from Pakistan and Egypt. The two studies with the largest enrolments were community-based. In two studies, cases were more than controls. There were wide variations in the primary outcomes, diagnostic criteria and putative risk factors explored across studies. In the three prospective cohort studies [25,30,31] included in the meta-analysis, follow-up was within 7 postnatal days and the reported attrition rate was less than the 20% general rule of thumb [Song & Chung, 2010].

**Table 1:** Characteristics of included studies in the data syntheses.

First author, year [reference]	Country	Study design	Cases	Non-cases/ controls	Total sample	Primary outcome	Diagnostic criterion	Risk factors	Covariates
**Sodeinde O, 1995 [21] (Apr 1989—Apr 1991)**	Nigeria	Case-control Preterm/Term	327	60	387	NNJ	Serum bilirubin >205μmol/L	G6PD, Serum aflatoxin	ABO, Rhesus disease, Birth weight
**Arif, 1999 [22] (Jan 1992—Dec 1994)**	Pakistan	Retrospective Cohort Mean age: 37.2wks	27	842	869	NNJ + ET	Not stated for ET. Significant NNJ defined as jaundice requiring phototherapy and/or exchange transfusion within the first seven days of life.	ABO, Rh disease, G6PD, Sepsis, Hypothermia	History of NNJ, Oxytocin, Birth asphyxia, Low birth weight
**Murki, 2001 [23] (July 1998—June 1999)**	India	Cross-sectional Age: ≥37wks	14	50	64	Kernicterus	Stage II bilirubin encephalopathy, i.e., presence of opisthotonus, rigidity and sun-setting of eyeballs.	Birth asphyxia, Max TSB, Free bilirubin	Gender, Gestational age, Birth weight, SFD, pH, Exclusive breastfeeding, Weight loss,
**Agrawal, 2009 [24] (June 2006—June 2007)**	India	Nested case-control Age: ≥35wks	77	50	127	Significant NNJ	Total serum bilirubin ≥ 18 mg/dL	Variant promoter UGT1A1, G6PD deficiency, History of treated NNJ in sibling	Gestational age, Gender, Oxytocin, ABO,
**Kalakheti, 2009 [25] (July 2002—June 2003)**	Nepal	Prospective Cohort ≥37wks: 186, <37wks: 14	37	163	200 [Loss to follow-up: 0%]	Significant NNJ	Serum bilirubin: >4 mg/dL at cord blood, >10 mg/dL at 24 hrs, >12 mg/dL at 48hrs and >15 mg/dL at 72 hrs	ABO	Ethnicity, Maternal age, Gestational age, Birth weight, Baby size (weight for gestational age), Sepsis
**Olusanya, 2009 [26] (July 2005—June 2007)**	Nigeria	Cross-sectional* ≥37wks: 5169, <37wks: 86	291	4,971	5,262	NNJ + PT	Parental history	Religion, Occupation, Herbal drug in pregnancy, Gender (Male), Underweight, Multiple gestation, Place of delivery	Maternal age, Ethnicity, Marital status, Parity Education, Social class, House ownership and type, Antenatal care, Gestational age, Attendants at birth, Mode of delivery, Cord cutting, Delayed cry at birth, Hospitalization in the first 28 days.
			98	5,164	5,262	NNJ + ET	Parental history	Religion, Herbal drug use in pregnancy, Gender (Male), Underweight & Place of delivery	
**Adebami, 2011 [27] (Jan 2007—Dec 2009)**	Nigeria	Cross-sectional BWT <2.5kg: 319; BWT ≥2.5kg	28	854	882	ABE	Clinical signs of ABE: decreased alertness/lethargy, high-pitched cry, hypotonia, fever, poor feeding or hypertonia of the extensor muscles, opisthotonus, abnomal movements	Place of birth, Weight on admission, Maternal age & Social class.	Gender, Outcome (not defined)
**Gamaledin, 2011 [28] (Jan 2008—Dec 2008)**	Egypt	Cross-sectional >34wks	44	205	249	ABE or BE	TSB ≥25 mg/dL plus clinical signs of moderate to severe ABE indexed by BIND score (4–9)	TSB level, Weight on admission, Rh disease, Sepsis	ABO
**Ogunlesi, 2011 [29] (Jan 2008—Dec 2009)**	Nigeria	Cross-sectional ≥37wks	75	77	152	ABE	TSB ≥15 md/dL plus clinical signs of ABE: poor sucking, exaggerated startle reaction, high-pitched cry, abnormal limb movements, hypotonia, hypertonia, retrocollis, opisthotonus or seizures	Out-born, Social class, Severe anemia, Acidosis	BWT, Maternal education, Delay in presentation ≥48hrs, Hypoglycemia
**Chawla, 2012 [30] (Aug—Oct 2009)**	India	Prospective Cohort ≥35wks & ≥2.0kg	65	327	462 [Loss to follow-up: 70 or 15.1%]	Pathological NNJ	Jaundice requiring phototherapy and/or exchange transfusion based on the high risk zone of AAP guideline up to 7th day.	Primiparity, TcB level, Gestational age,	Prolonged rupture of membranes, Oxytocin infusion, Age of first passage of meconium.
**Kaur, 2012 [31] (Feb—June 2010)**	India	Prospective Cohort ≥35wks or ≥2.0kg	199	732	997 [Loss to follow-up: 66 or 6.6%]	Pathological NNJ	Jaundice requiring phototherapy and/or exchange transfusion based on the middle or lower line of AAP guideline up to 7th day.	Pre-discharge TcB, Gestational age	Parity, Sibling with treated NNJ, Birth weight, Supplemental feeding within 24hrs of birth, Bruises, Cephalhematoma
**Scrafford, 2013 [32] (May 2003—Jan 2006)**	Nepal	Retrospective Cohort* ≥37wks: 15,518, <37wks: 3454 Unknown: 13	556	18,429	18,985	Significant NNJ	Visual assessment based on report of ‘yellow body/eyes’ over a median of 10 days of follow-up.	Primiparity, Ethnicity, Prolonged labor, Improved latrine, Ambient air temperature, Oil massage, Gender (Male), Birth weight, Difficulty feeding.	Maternal age, Parental education, Gestational age, SGA, Exclusive breastfeeding, Colostrum, Place of delivery, Skilled attendants at birth, Color at birth, Injury at birth, Multiple pregnancy, Vaginal bleeding, convulsions or fever 7 days before delivery, Electricity, Television
**Tiwari, 2014 [33] (3ys, dates not stated)**	India	Matched Case-Control Inborn: (37–41wks), Out-born: ≤2wks	113	218	331	Significant NNJ	Visual assessment. TSB estimation was selective. TSB >95th percentile on AAP nomogram, Controls: TSB <75th percentile	Weight loss, Sepsis, ABO, CAT insertion, UGT1A1 (c.211G>A variant & g.-3279 T>G variant), TATA box polymorphism	Sibling treated for NNJ, Hypothyroidsm

*Community-based, NNJ: neonatal jaundice, ABE: acute bilirubin encephalopathy, BE: bilirubin encephalopathy, AAP: American Academy of Pediatrics, PT: phototherapy,

ET: exchange transfusion, TcB: transcutaneous bilirubin, TSB: total serum bilirubin, BWT: birth weight, G6PD: glucose-6-phosphate dehydrogenase, SGA: Small-for-gestational age

Assessment of the methodological qualities of the included studies is presented in S3 Table. The risk of bias was low in nine studies and medium in four studies. Only two studies were community-based. The major weakness with the community-based studies was that case definition was either based on parental history or visual assessment by the health workers. Statistical determination of sample size *a priori* was not reported in any study.

### Qualitative synthesis

A total of five countries, India, Nigeria, Pakistan, Nepal and Egypt contributed to the data used in this study. These five countries have a combined annual live births of approximately 41 million, accounting for 30% of the global annual live births of roughly 135 million. Four studies reported the risk factors for bilirubin-induced neurologic dysfunction (ABE and/or kernicterus) while the primary outcome in the remaining nine studies was severe hyperbilirubinemia requiring phototherapy or exchange transfusion. The range of maternal, prenatal and neonatal factors found to be significantly or not significantly associated with severe hyperbilirubinemia are summarized in Table 2. There was a wide variation in the number and type of factors explored across the 13 studies. Several factors that are rarely reported in high-income countries, were documented in single studies such as: religion, maternal occupation, social class and herbal drug use in pregnancy. Some factors such as race, rhesus disease, ABO incompatibility, maternal age, social class, primiparity, male gender, sepsis, sibling treated for jaundice, gestational age, low birth weight and weight loss that were found to be associated with severe hyperbilirubinemia in some studies were not found to have such association in other studies. Genetic factors such as G6PD deficiency and (TA)_n_ promoter polymorphism of the urine-diphosphate-glucuronosyltransferase 1A1 gene (UGT1A1) were not reported in any studies as having no association with severe hyperbilirubinemia. No study investigated the association between visible jaundice in the first 24 hours of life and severe hyperbilirubinemia.

**Table 2:** Summary of reported risk factors for severe neonatal hyperbilirubinemia*.

Category	Factors	Studies with positive finding	Studies with negative finding^+^
**Maternal/Family**	Race or Ethnicity	32	25,26
	Rhesus disease	22,28	21
	ABO incompatibility	22,25,33	21,24,28
	Oxytocin during labor		22,24,30
	Exclusive breastfeeding		23,32
	Religion	26	
	Occupation	26	
	Maternal age	27	25,26,32
	Social class	27,29	26
	Primiparity	30,32	26,31
	Herbal drug in pregnancy	26	
	Prolonged labor	32	
	Place of delivery	26,27,29	32
	Family history of jaundice		22
	Sibling treated for jaundice	24	31,33
**Perinatal**	Birth trauma		31
	Male gender	32	23,24,27,
	Infections	22,28,33	25
	Birth asphyxia	23	22
	Multiple gestation	26	32
	Severe anemia	29	
	Acidosis	19	
**Neonatal**	Preterm birth/gestational age	30,31	21,23,24,25,26,32
	Low birth weight	32	22,23,25,29,31
	Hypothermia	22	
	TcB/TSB level	23,28,30,31	
	Free bilirubin	23	
	Serum aflatoxin	21	
	Underweight/weight loss	26,27,28,33	23,25,32
	G6PD deficiency	21,22,24	
	UGT1A1 Gene Polymorphisms	24,33	

*inclusive of acute bilirubin encephalopathy and kernicterus;

^+^Limited to factors reported in eligible studies.

TcB: transcutaneous bilirubin, TSB: total plasma/serum bilirubin, G6PD: glucose-6-phosphate dehydrogenase,

### Quantitative synthesis

**Fig 2:**
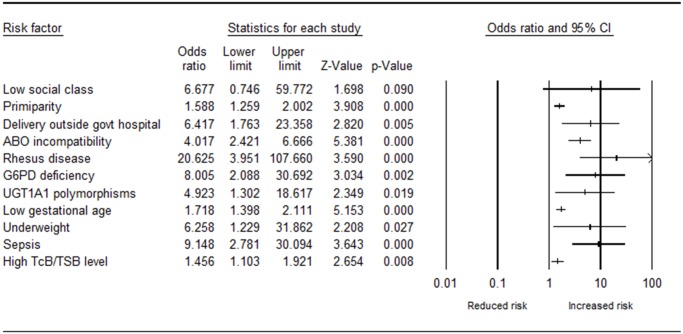
Forest plot of risk factors for severe neonatal hyperbilirubinemia in low and middle income countries.

All the 13 studies were included in the meta-analysis. All primary outcomes with or without ABE or kernicterus were considered as severe hyperbilirubinemia. A total of eleven risk factors were reported in two or more studies and included in the meta-analysis (Table 3 and Fig. 2). Additional details about the individual studies included in the pooled estimate for each of the risk factors are provided in the supplementary data (S1 Fig.). Five of the risk factors are maternal and six are neonatal.

**Table 3:** The meta-analysis results of selected risk factors for severe neonatal hyperbilirubinemia.

Risk factors	No of studies [references]	No. of subjects	Pooled OR (95% CI)	Z	p-value	Heterogeneity		P_Egger’s_
						I^2^ (%)	p-value	
***Maternal***								
**Social class**	2 [27,29]	1034	6.68 (0.75–59.78)	1.70	0.090	98.6	<0.0001	-
**Primiparity**	2 [30,32]	19469	1.59 (1.26–2.00)	3.90	<0.0001	0	0.352	-
**Place of delivery**	3 [26,27,29]	6296	6.42 (1.76–23.36)	2.82	0.005	92.5	<0.0001	0.376
**ABO incompatibility**	3 [22,25,33]	1400	4.02 (2.42–6.67)	5.38	<0.0001	2.7	0.358	0.680
**Rhesus disease**	2 [22,28]	1118	20.63 (3.95–107.65)	3.59	<0.0001	73.4	0.053	-
***Neonatal***								
**G6PD deficiency**	3 [21,22,24]	1383	8.01 (2.09–30.69)	3.03	0.002	64.4	0.060	0.052
**UGT1A1 polymorphisms**	2 [24,33]	458	4.92 (1.30–18.62)	2.35	0.019	74	0.049	-
**Gestational age**	2 [30,31]	1481	1.71 (1.40–2.11)	5.16	<0.0001	0	0.701	-
**Underweight/weight loss**	4 [26,27,28,33]	6724	6.26 (1.23–31.86)	2.21	0.027	99.1	<0.0001	0.352
**Sepsis**	3 [22,28,33]	1449	9.15 (2.78–30.1)	3.64	<0.0001	42.7	0.174	0.600
**TcB/TSB level**	4 [23,28,30,31]	1794	1.46 (1.10–1.92)	2.65	0.008	95.8	<0.0001	0.077

The maternal factors included in the meta-analysis were social class status, parity, place of delivery, ABO incompatibility and rhesus incompatibility. Two studies from Nigeria [27,29] examined the role of social class and severe hyperbilirubinemia but the pooled data did not show any significant association (p = 0.090). Two studies from India [30] and Nepal [32] examined the role of parity on the risk of severe hyperbilirubinemia. The pooled data indicated that infants born to primiparous mothers were at increased risk of severe hyperbilirubinemia (OR, 1.59; 95% CI, 1.26–2.00, p<0.0001), with low heterogeneity (I^2^ = 0.0%). Three studies all from Nigeria [26,27,29] investigated the role of the place of delivery on the risk of hyperbilirubinemia. The pooled data revealed that infants born outside hospital, especially government-owned hospital, were at increased risk of severe hyperbilirubinemia (OR, 6.42; 95% CI, 1.76–23.36, p = 0.005), with high heterogeneity (I^2^ = 92.5%). The pooled data of three studies from Pakistan [22], Nepal [25] and India [33] that examined ABO incompatibility showed an increased risk of severe hyperbilirubinemia (OR, 4.01; 95% CI, 2.44–6.61, p<0.0001), with low heterogeneity (I^2^ = 2.7%). Two studies from Pakistan [22] and Egypt [28] analyzed the role of rhesus incompatibility on the risk of neonatal jaundice and the pooled data indicated that infants with rhesus disease were at increased risk of severe hyperbilirubinemia (OR, 20.63; 95% CI, 3.95–107.65, p<0.0001), with high heterogeneity (I^2^ = 73.4%).

The six neonatal factors with sufficient data for the meta-analysis were G6PD deficiency, UGT1A1 polymorphisms, gestational age, weight on admission, sepsis and TcB/TSB levels. Three studies from Nigeria [11], India [24] and Pakistan [22] examined the role of G6PD deficiency on the risk of neonatal hyperbilirubinemia. The pooled data showed that infants with G6PD deficiency have an elevated risk of severe hyperbilirubinemia (OR, 8.01; 95% CI, 2.09–30.69, p = 0.002), with high heterogeneity (I^2^ = 64.4%). The pooled data of the two studies from India [24,33] that explored the contribution of UGT1A1 polymorphisms to the risk of neonatal hyperbilirubinemia showed significant cumulative risk estimate (OR, 4.92; 95% CI, 1.30–18.62, p = 0.019), with high heterogeneity (I^2^ = 74.0%). Gestational age in relation to the risk of severe hyperbilirubinemia in two studies from India [30,31] and the pooled data showed that infants with low gestational age (<37 weeks) were at increased risk of severe hyperbilirubinemia (OR, 1.71; 95% CI, 1.40–2.11, p<0.0001), with low heterogeneity (I^2^ = 0.0%). Four studies from Nigeria [26,27], Egypt [28] and India [33] examined the role of infant weight on admission for severe jaundice. The pooled data showed infants who were underweight or with weight loss on admission were at increased risk of severe hyperbilirubinemia (OR, 6.26; 95% CI, 1.23–31.86, p = 0.027), with high heterogeneity (I^2^ = 99.1%). Three reports from Pakistan [22], Egypt [28], and India [33], studied the role of sepsis on the risk of severe hyperbilirubinemia. The pooled data indicated that infants diagnosed with sepsis were at increased risk of severe hyperbilirubinemia (OR, 9.15; 95% CI, 2.78–30.1, p<0.0001), with moderate heterogeneity (I^2^ = 42.7%). Four studies from India [23,30,31] and Egypt [28] explored the impact of various bilirubin levels on the risk of severe hyperbilirubinemia. The pooled data showed that infants with elevated TcB and/or TSB levels, adjusted for gestational age, were at increased risk of severe hyperbilirubinemia (OR, 1.46; 95% CI, 1.10–1.92, p<0.0001), with high heterogeneity.

Other risk factors reported in single studies are shown in Table 2. They include maternal/family factors such as maternal age [27], race/ethnicity [32], religion [26], occupation [26], herbal drug use in pregnancy [26], prolonged labor [32] and sibling treated with jaundice [24]. Perinatal and neonatal factors include gender [32], birth asphyxia [23], multiple gestation [26], severe anemia [29], acidosis [19], low birth weight [32], hypothermia [22], free bilirubin [23], and serum aflatoxin [21].

### Publication bias

There was no evidence of publication bias in the pooled estimates for risk factors involving three or more studies based on the results of Egger’s test (p>0.05) as shown in Table 3. Publication bias could not be assessed in pooled data involving only two studies. In the sensitivity analysis, no significant changes were evident in the observed directions and effect sizes following the random removal of a single study in each turn. We could not explore the funnel plot symmetry because of the small number of studies.

## Discussion

For the first time, there is a growing recognition among leading policy research groups such as the Child Health Epidemiology Reference Group (CHERG) of the World Health Organization (WHO) and the Global Burden of Disease Collaborators of the clinical and public health significance of hyperbilirubinemia in newborns as an important neonatal condition that deserves global health attention in the post-2015 millennium development goals era [35,36]. Available, albeit limited, evidence suggests that the burden of severe neonatal hyperbilirubinemia is greatest in LMICs and underscores the unique contribution of this systematic review in providing insights into the risk factors that need to be addressed in curtailing this burden. To our best knowledge, this study is the first to undertake and report a systematic review and meta-analysis of the risk factors for severe hyperbilirubinemia, especially in the most economically disadvantaged populations in LMICs. While a number of maternal and neonatal factors have been identified for possible intervention, the overarching finding is the need to undertake more robust epidemiological studies covering a wide range of putative demographic, biological and clinical risk factors. For example, majority of the studies included in our review were hospital-based and were in many instances significantly under-powered to determine real differences between subjects and controls. Some of the factors were only reported in single studies, making it difficult to generalize their findings in the country of study or to other LMICs. Additionally, data from 86 out of the 91 eligible LMICs were lacking, while the five countries with data used in this review account for about 47% of the 86.7million annual live births in the 91 eligible LMICs.

Prevention of these risk factors or identifying infants with these risk factors is a crucial first step in effectively managing infants with or at risk of severe hyperbilirubinemia [7,8]. The risks associated with maternal factors such as primiparity and place of delivery can be addressed through improved maternal and public health education. Blood group incompatibilities (ABO & Rhesus disease) can be addressed through routine antenatal care and identification of mothers whose babies may be at risk of these disorders and in turn require surveillance for the development of severe hyperbilirubinemia [9,37]. In one report by Bhutani et al, the global prevalence of Rh hemolytic disease worldwide was estimated at 276/100,000 live births, translating to 373,300 babies in 2010 [9]. Besides Europe/Central Asia, South Asia and Sub-Saharan Africa were found to have the highest prevalence, estimated at roughly 386/100,000 live births [9]. This is in contrast to an estimated prevalence of 2.5/100,000 live births in high-income countries with well-established health-care systems that offer advanced perinatal-neonatal care for pregnant mothers.

While the precise causal mechanism between G6PD deficiency and hyperbilirubinemia is not yet fully understood, early detection of G6PD deficient infants is essential to effectively manage the risk of severe hyperbilirubinemia in the affected infants [38,39]. The subsisting recommendation of a WHO Working Group is that population screening of all newborn babies should be implemented in areas with a prevalence of G6PD deficiency of 3–5% or more in males [40]. At least 33 countries of the eligible LMICs in this review have a national prevalence in excess of 10% for G6PD deficiency [41]. While universal screening of all newborns would seem impracticable immediately in many settings due to resource constraints, such screening should be routinely provided in all secondary and tertiary points-of-care for neonatal jaundice [42]. Some cost-effective tools for accomplishing this intervention have been demonstrated in several resource-limited countries [43,44]. Some studies suggest that hyperbilirubinemia may be exacerbated in ethnic African populations where UGT1A1 polymorphisms associated with Gilbert’s syndrome are prevalent, especially with concurrent G-6-PD deficiency [45]. Additionally, unconjugated bilirubin levels in infants with G6PD deficiency in combination with (TA)_n_ promoter polymorphism often rise exponentially from haemolysis triggered by exposure to oxidant stressors such as sepsis and menthol-based products [42,45].

This review also confirms the need to consider infants with low gestational age (<37 weeks), infection or elevated bilirubin levels in the first hours of life and monitor them appropriately. Routine laboratory investigation for sepsis and bilirubin levels should be standard of care for newborns presenting in hospitals. Prompt identification of underweight infants, with or without visual evidence of weight loss on admission, should also be incorporated into the clinical protocol for the management of neonatal hyperbilirubinemia in LMICs.

While this review complements our earlier work exploring the levels of delay experienced by infants with hyperbilirubinemia requiring treatment in LMICs [15], the overall quality deserves mention in view of the small number of included studies. Substantial heterogeneity was observed across majority of the studies included in the meta-analysis despite the use of random effects model and the selection of only observational studies with statistical adjustments for confounding. This observation was not unexpected given the differences in case definition of severe hyperbilirubinemia, primary outcomes, sample size, study design, population characteristics, range of covariates and adjustment for confounding factors. Despite the inherent weaknesses in the meta-analysis of observational studies, some authors have argued that some degree of heterogeneity is not without some value [46,47]. It improves the generalizability of the results of the meta-analysis particularly where such heterogeneity is carefully estimated, and the results are cautiously interpreted as in our study. The pooled estimates of odds ratios are also valuable and important indicators for assessing the risk factors of a disease or disorder, as they may facilitate the identification of factors that influence the outcome that were not observable in individual studies.

A number of additional limitations of this study are worth noting. Firstly, the meta-analysis was based on data drawn from only five countries which may affect the generalizability of some of the findings to other eligible LMICs. Secondly, potentially important risk factors were excluded because of lack of (insufficient or reliable) data. However, the absence of evidence is not necessarily evidence of absence of the significance of the excluded risk factors [48]. Thirdly, we broadened the definition of severe hyperbilirubinemia to include all severity types: mild, moderate, severe, extreme hyperbilirubinemia as well as acute and chronic bilirubin encephalopathy. However, risk factors may vary for various levels of severity of hyperbilirubinemia, ABE and kernicterus [8]. Fourthly, we were unable to determine regional differences in risk factors even among the three regions (South Asia, Sub-Saharan Africa and Middle-East/North Africa) covered in this study. Fifthly, the diagnostic criteria for the reported risk factors were not uniform across all studies. Lastly, the sources of heterogeneity could not be identified by methods such as meta-regression or subgroup analysis due to fewer number of studies. Notwithstanding, the clinical risk factors reported in this study are consistent with findings in several studies that have investigated the causes of severe neonatal hyperbilirubinemia in LMICs and thus warrant attention.

## Conclusions

Despite the limitation of the few countries and studies covered, this systematic review has shown that a range of maternal and neonatal factors that can be effectively addressed at all levels of health care delivery by available interventions continue to place infants in LMICs at increased risk of severe hyperbilirubinemia. The need for more robust epidemiological studies on the profile of infants with or at risk of severe hyperbilirubinemia across all the eligible LMICs is demonstrated by the findings in this report. The emerging recognition of hemolytic disease in fetus and newborn and other neonatal jaundice as separate and important disease category in the post-2015 global child health agenda offers an unprecedented opportunity to curtail the avoidable burden of severe hyperbilirubinemia, especially in the most developmentally disadvantaged LMICs.

## Supporting Information

S1 Fig Forest plot of low social class and risk of severe hyperbilirubinemia. (TIF)

S2 Fig Forest plot of primiparity and risk of hyperbilirubinemia. (TIF)

S3 Fig Forest plot of place of delivery and risk of severe hyperbilirubinemia. (TIF)

S4 Fig Forest plot of ABO incompatibility and risk of severe hyperbilirubinemia. (TIF)

S5 Fig Forest plot of Rhesus disease and risk of severe hyperbilirubinemia. (TIF)

S6 Fig Forest plot of G6PD deficiency and risk of severe hyperbilirubinemia. (TIF)

S7 Fig Forest plot of UGT1A1 polymorphisms and risk of severe hyperbilirubinemia. (TIF)

S8 Fig Forest plot of gestational age and risk of severe hyperbilirubinemia. (TIF)

S9 Fig Forest plot of underweight/weight loss and risk of severe hyperbilirubinemia. (TIF)

S10 Fig Forest plot sepsis and risk of severe hyperbilirubinemia. (TIF)

S11 Fig Forest plot of high TcB/TSB levels and risk of severe hyperbilirubinemia. (TIF)

S1 PRISMA Checklist (DOCX)

S1 Table Eligible low and middle-income countries (per capita gross national income ≤$6,000). (PDF)

S2 Table Quality assessment checklist for selected studies. (DOCX)

S3 Table Methodological quality of included studies. (DOCX)
