# Supplementary figures and images for: Risk Factors for Severe Neonatal Hyperbilirubinemia in Low and Middle-Income Countries: A Systematic Review and Meta-Analysis
Source: PLoS One. 2015 Feb 12;10(2):e0117229. doi: 10.1371/journal.pone.0117229 (PMC4326461; doi:10.1371/journal.pone.0117229)

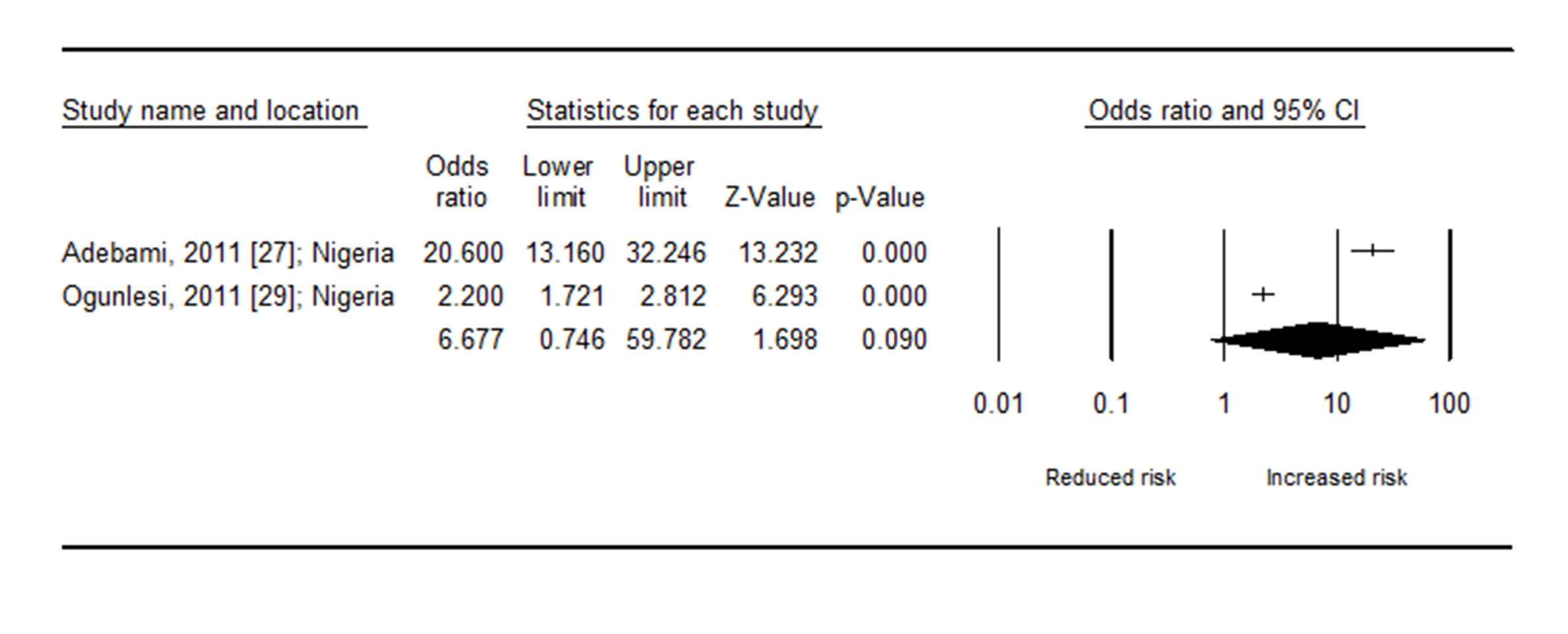

Supplement: S1 Fig — (TIF) [file pone.0117229.s001.tif]

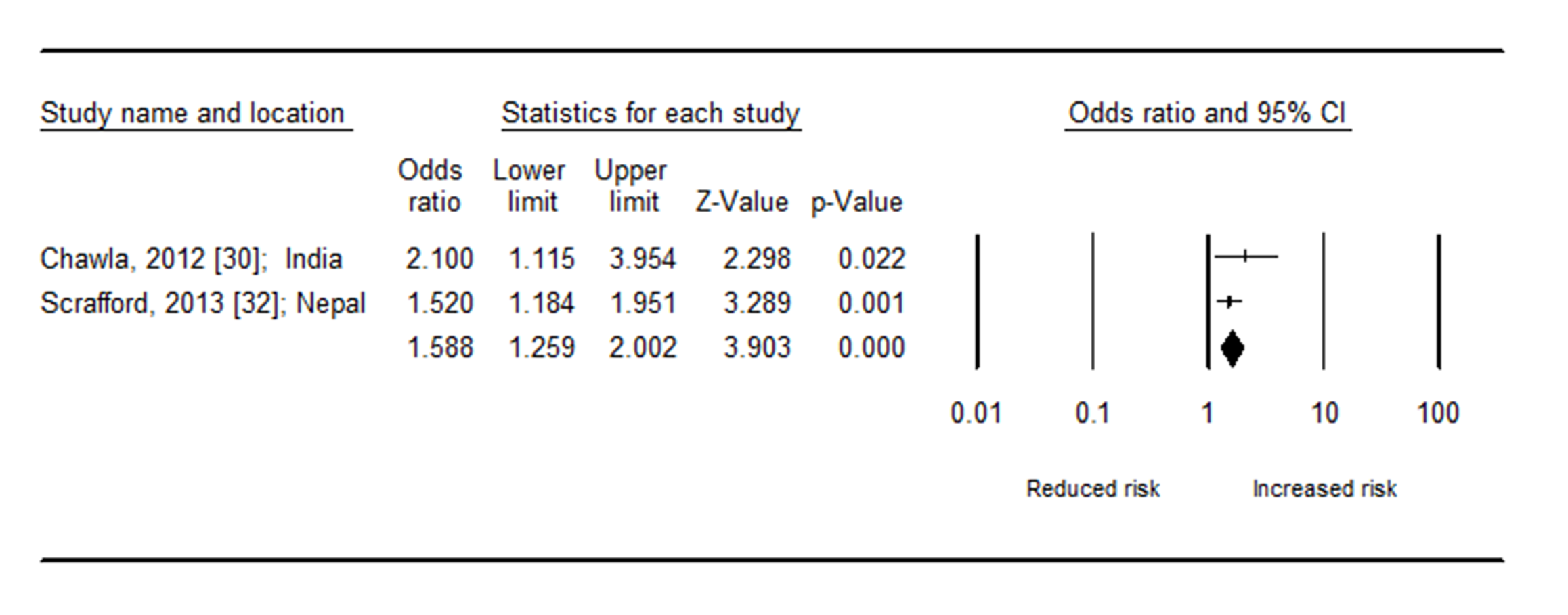

Supplement: S2 Fig — (TIF) [file pone.0117229.s002.tif]

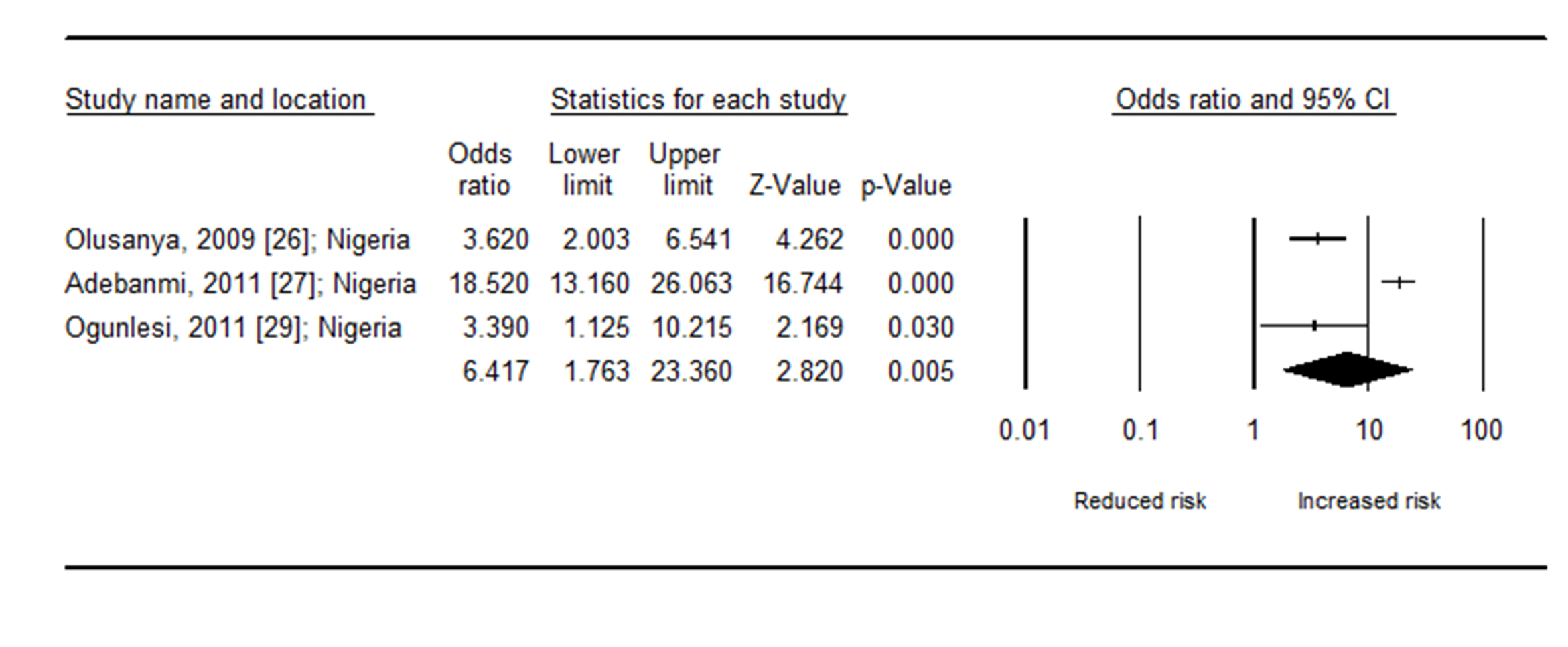

Supplement: S3 Fig — (TIF) [file pone.0117229.s003.tif]

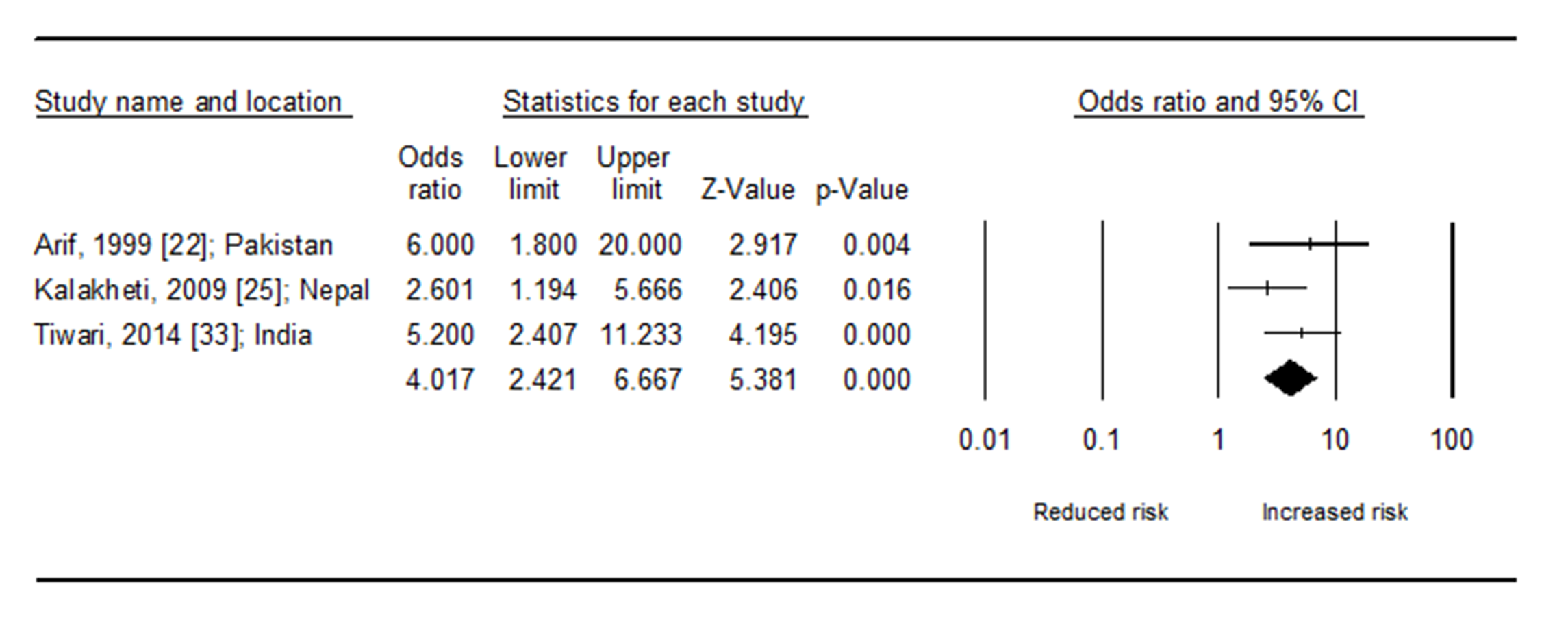

Supplement: S4 Fig — (TIF) [file pone.0117229.s004.tif]

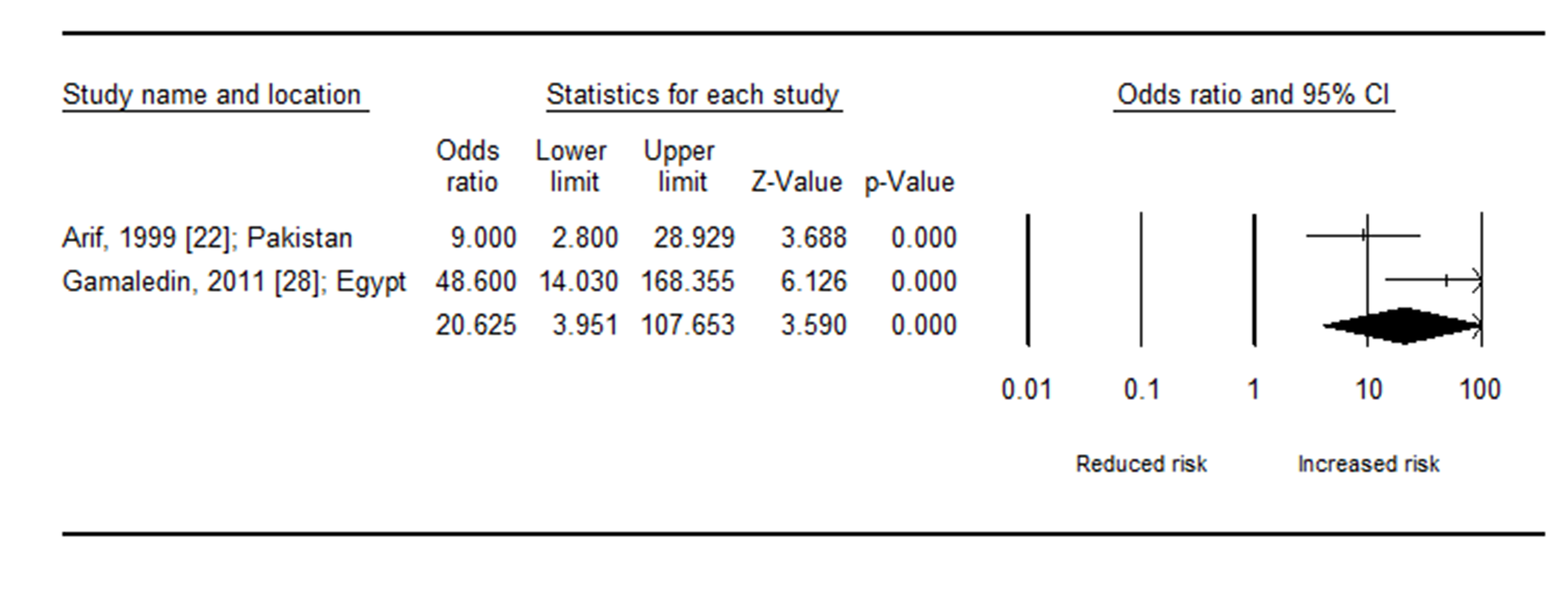

Supplement: S5 Fig — (TIF) [file pone.0117229.s005.tif]

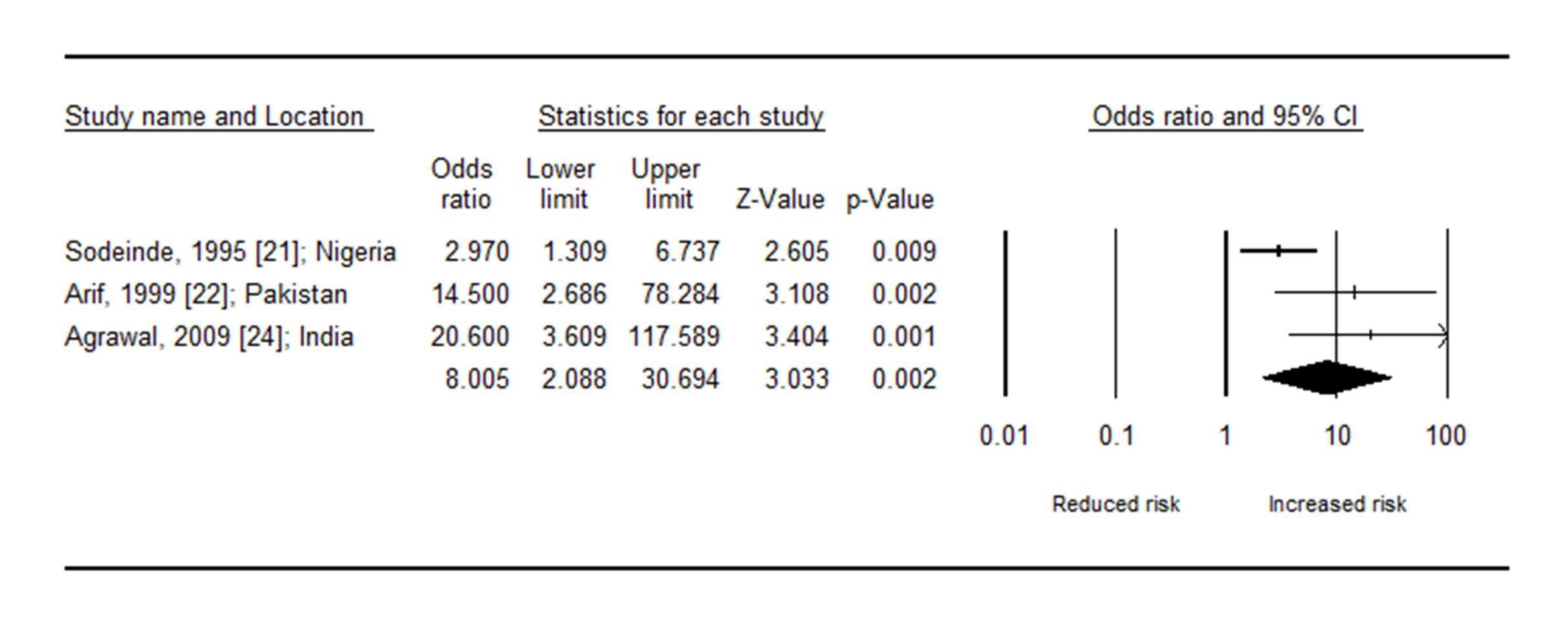

Supplement: S6 Fig — (TIF) [file pone.0117229.s006.tif]

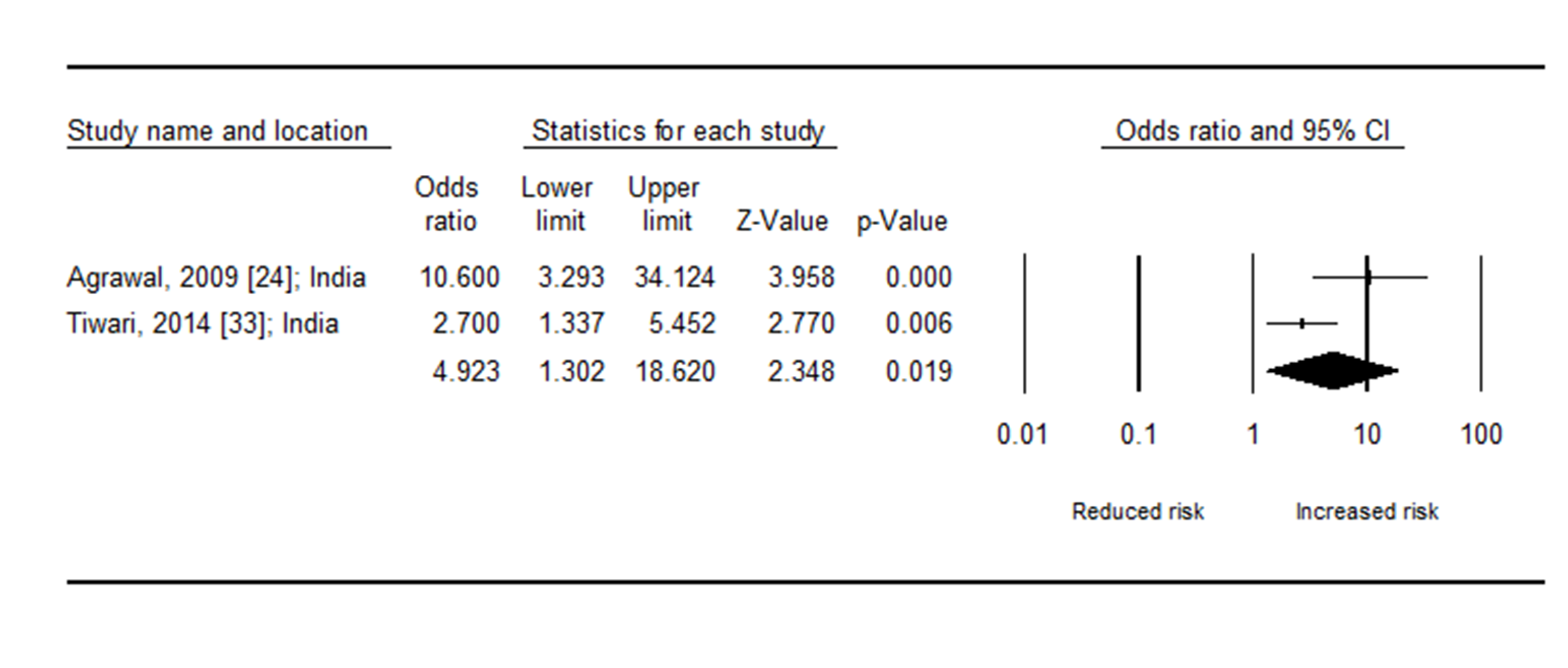

Supplement: S7 Fig — (TIF) [file pone.0117229.s007.tif]

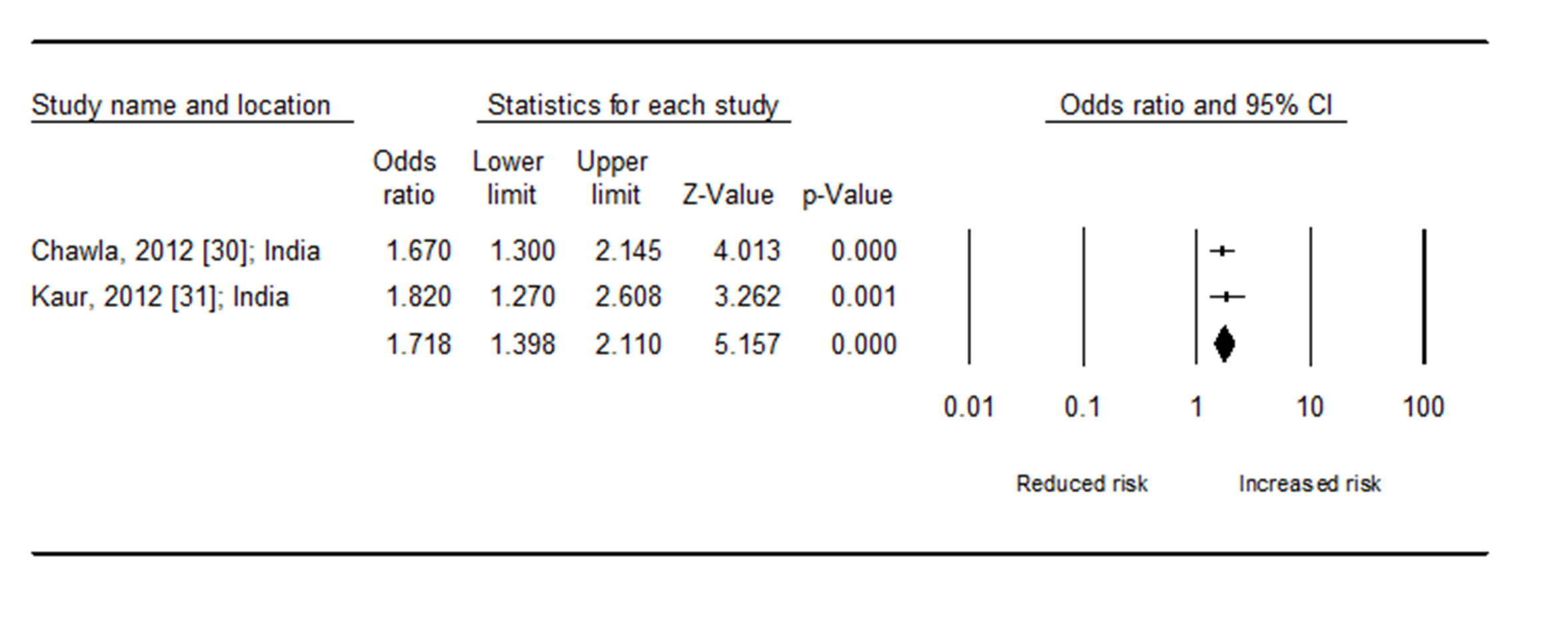

Supplement: S8 Fig — (TIF) [file pone.0117229.s008.tif]

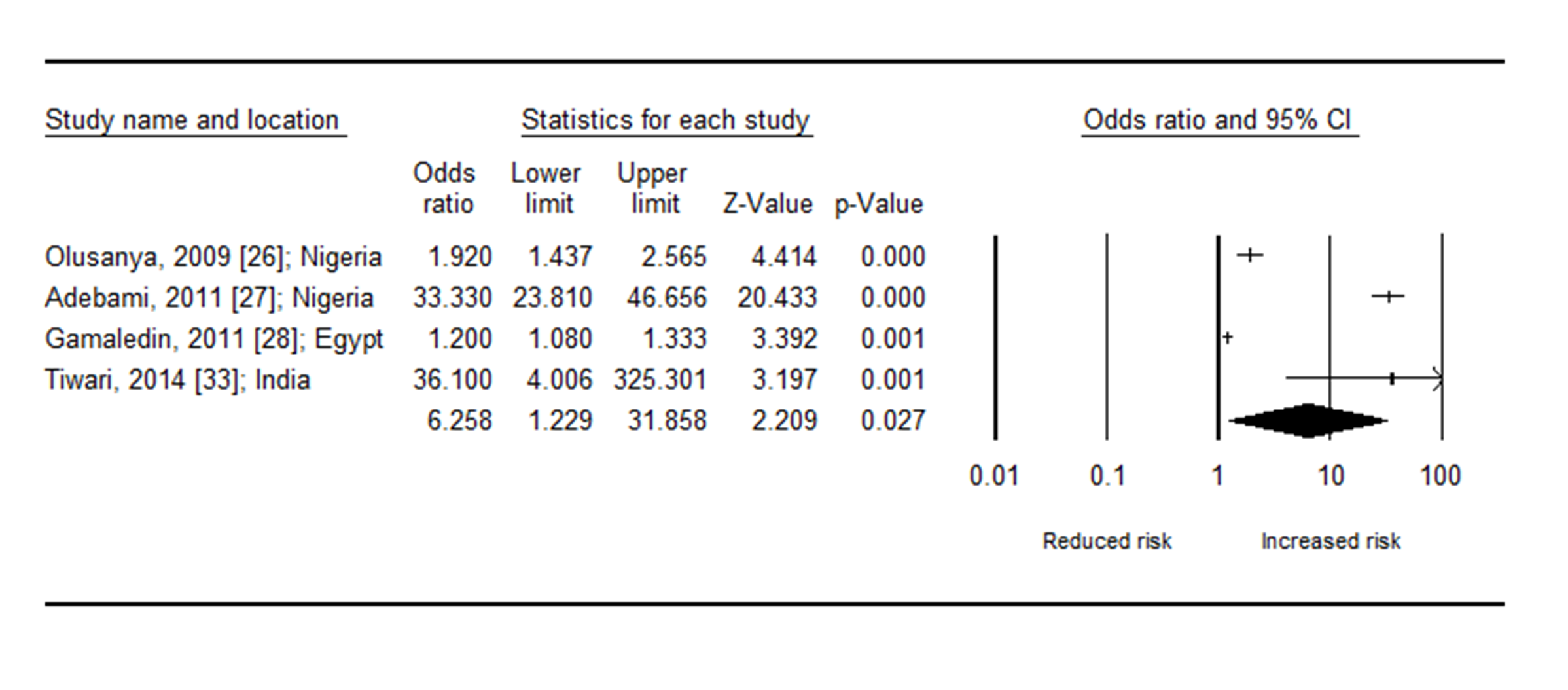

Supplement: S9 Fig — (TIF) [file pone.0117229.s009.tif]

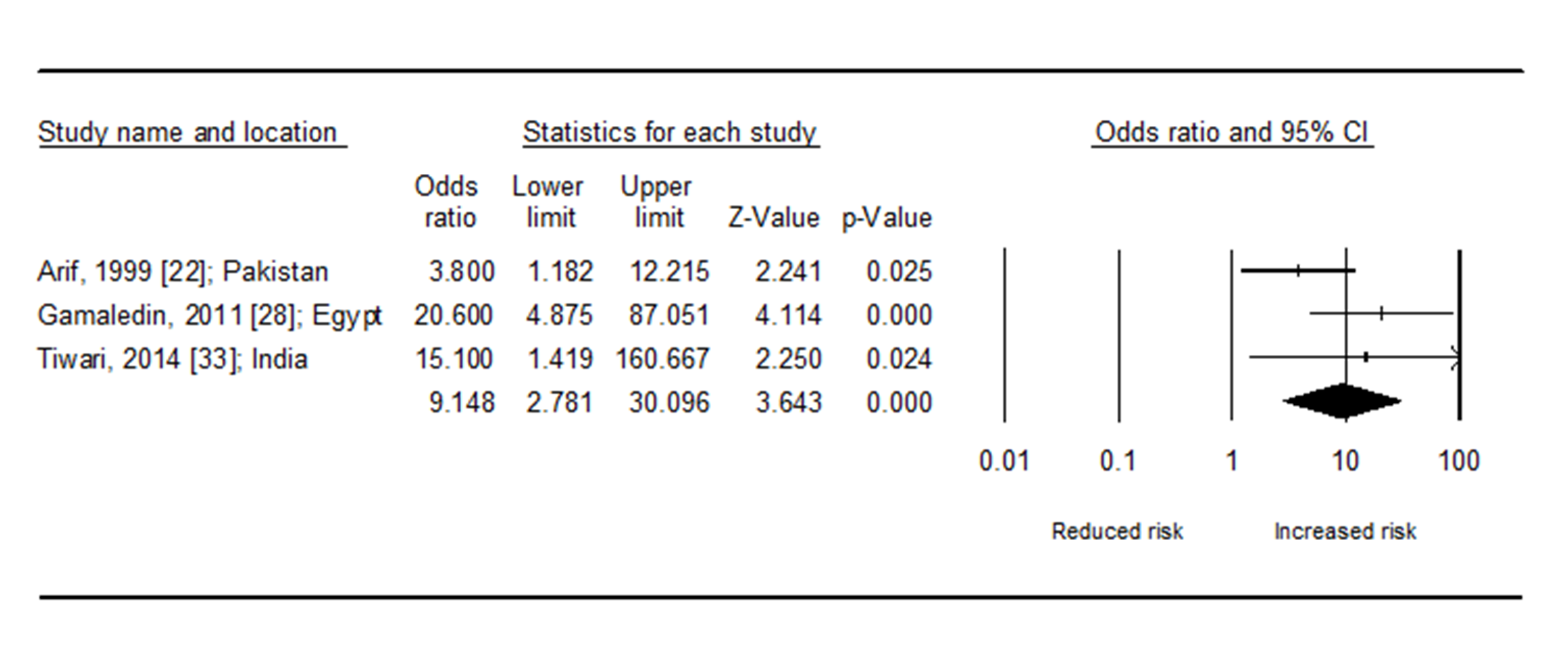

Supplement: S10 Fig — (TIF) [file pone.0117229.s010.tif]

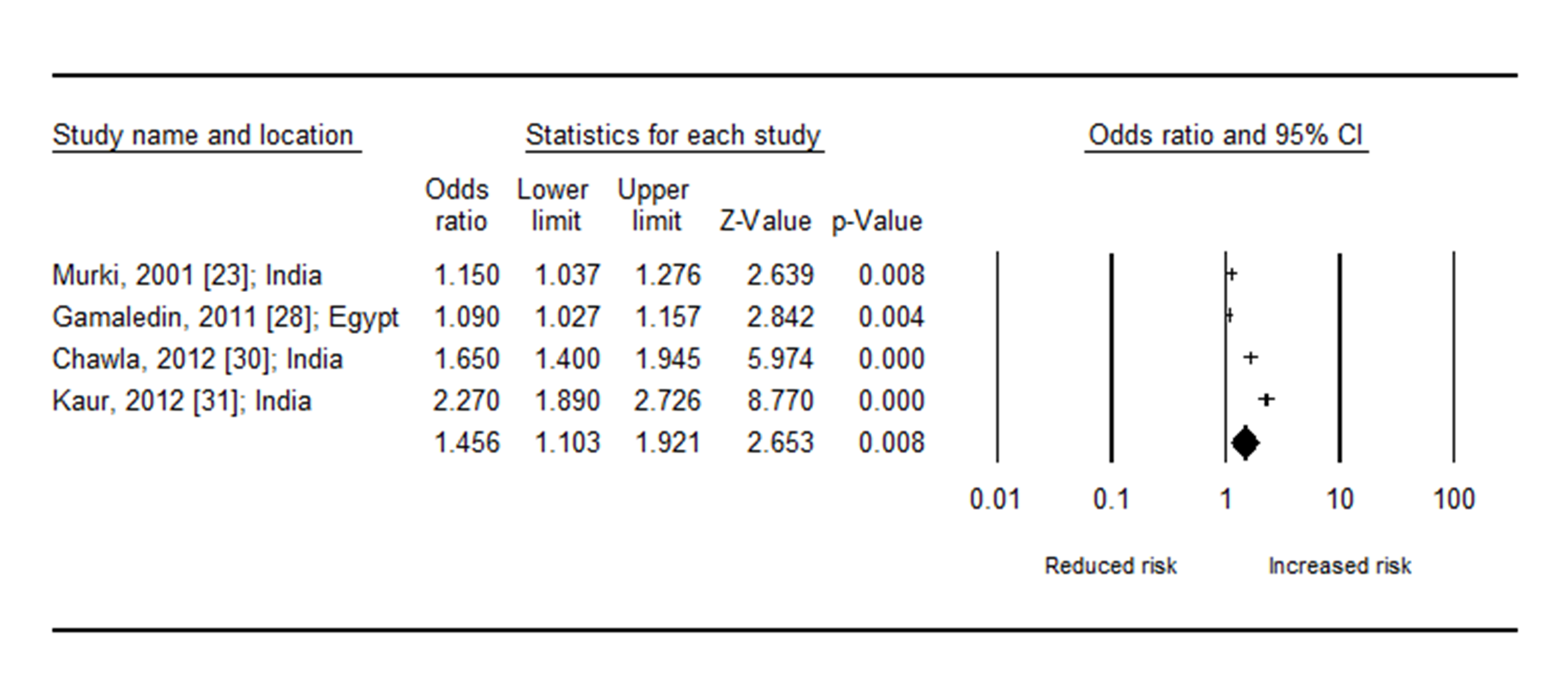

Supplement: S11 Fig — (TIF) [file pone.0117229.s011.tif]
