# Supplementary material for: Risk Factors for Severe Neonatal Hyperbilirubinemia in Low and Middle-Income Countries: A Systematic Review and Meta-Analysis
Source: PLoS One. 2015 Feb 12;10(2):e0117229. doi: 10.1371/journal.pone.0117229 (PMC4326461; doi:10.1371/journal.pone.0117229)
Supplement: S1 Table — (PDF) [file pone.0117229.s013.pdf]

Table S1. Eligible low and middle-income countries (GNI per capita ≤$6,000)

SN	COUNTRY	Region	HDI RANK 2012	HDI	Life Expectancy (Years)	GNI per capita ($)	Annual Livebirths ('000)	Hospital Delivery (%)
1	Afghanistan	SOA	175	0.374	49.1	1,000	1,408	33
2	Angola	SSA	148	0.508	51.5	4,812	803	46
3	Armenia	ECA	87	0.729	74.4	5,540	47	99
4	Bangladesh	SOA	146	0.515	69.2	1,785	3,016	29
5	Belize	LAC	96	0.702	76.3	5,327	8	89
6	Benin	SSA	166	0.436	56.5	1,439	356	87
7	Bhutan	SOA	140	0.538	67.6	5,246	15	63
8	Bolivia, Plurinational State of	LAC	108	0.675	66.9	4,444	264	68
9	Burkina Faso	SSA	183	0.343	55.9	1,202	730	66
10	Burundi	SSA	178	0.355	50.9	544	288	60
11	Cambodia	EAP	138	0.543	63.6	2,095	317	54
12	Cameroon	SSA	150	0.495	52.1	2,114	716	61
13	Cape Verde	SSA	132	0.586	74.3	3,609	10	76
14	Central African Republic	SSA	180	0.352	49.1	722	156	53
15	Chad	SSA	184	0.34	49.9	1,258	511	16
16	Comoros	SSA	169	0.429	61.5	986	28	
17	Congo	SSA	142	0.534	57.8	2,934	145	92
18	Congo, Democratic Republic of the	SSA	186	0.304	48.7	319	2,912	75
19	Côte d'Ivoire	SSA	168	0.432	56	1,593	679	57
20	Cuba	LAC	59	0.78	79.3	5,539	110	100
21	Djibouti	MEN	164	0.445	58.3	2,350	26	87
22	Egypt	MEN	112	0.662	73.5	5,401	1,886	72
23	El Salvador	LAC	107	0.68	72.4	5,915	126	85
24	Eritrea	SSA	181	0.351	62	531	193	26
25	Ethiopia	SSA	173	0.396	59.7	1,017	2,613	10
26	Fiji	EAP	96	0.702	69.4	4,087	18	
27	Gambia	SSA	165	0.439	58.8	1,731	67	56
28	Georgia	ECA	72	0.745	73.9	5,005	51	98
29	Ghana	SSA	135	0.558	64.6	1,684	776	67
30	Guatemala	LAC	133	0.581	71.4	4,235	473	51
31	Guinea	SSA	178	0.355	54.5	941	394	39
32	Guinea-Bissau	SSA	176	0.364	48.6	1,042	59	42
33	Guyana	LAC	118	0.636	70.2	3,387	13	89
34	Haiti	LAC	161	0.456	62.4	1,070	266	25
35	Honduras	LAC	120	0.632	73.4	3,426	205	67
36	India	SOA	136	0.554	65.8	3,285	27,098	47
37	Indonesia	EAP	121	0.629	69.8	4,154	4,331	55
38	Iraq	MEN	131	0.59	69.6	3,557	1,144	65
39	Jordan	MEN	100	0.7	73.5	5,272	154	99
40	Kenya	SSA	145	0.519	57.7	1,541	1,560	43
41	Kiribati	EAP	121	0.629	68.4	3,079	22	66
42	Kyrgyzstan	ECA	125	0.622	68	2,009	131	97
43	Lao People's Democratic Republic	EAP	138	0.543	67.8	2,435	140	17
44	Lesotho	SSA	158	0.461	48.7	1,879	60	59
45	Liberia	SSA	174	0.388	57.3	480	157	37
46	Madagascar	SSA	151	0.483	66.9	828	747	35
47	Malawi	SSA	170	0.418	54.8	774	686	73
48	Mali	SSA	182	0.344	51.9	853	728	45
49	Marshall Islands	EAP		..	72.3	4,040	27	85
50	Mauritania	SSA	155	0.467	58.9	2,174	118	48
51	Micronesia, Federated States of	EAP	117	0.645	69.2	3,352	3	
52	Moldova, Republic of	ECA	113	0.66	69.6	3,319	44	99
53	Mongolia	EAP	108	0.675	68.8	4,245	65	99
54	Morocco	MEN	130	0.591	72.4	4,384	620	73
55	Mozambique	SSA	185	0.327	50.7	906	889	58
56	Myanmar	EAP	149	0.498	65.7	1,817	824	36
57	Namibia	SSA	128	0.608	62.6	5,973	60	81
58	Nepal	SOA	157	0.463	69.1	1,137	722	35
59	Nicaragua	LAC	129	0.599	74.3	2,551	138	74
60	Niger	SSA	186	0.304	55.1	701	777	17
61	Nigeria	SSA	153	0.471	52.3	2,102	6,458	35
62	Pakistan	SOA	146	0.515	65.7	2,566	4,764	41
63	Palestine, State of	MEN	110	0.67	73	3,359	33	
64	Papua New Guinea	EAP	156	0.466	63.1	2,386	208	52
65	Paraguay	LAC	111	0.669	72.7	4,497	158	82
66	Philippines	EAP	114	0.654	69	3,752	2,358	44
67	Rwanda	SSA	167	0.434	55.7	1,147	449	69
68	Samoa	EAP	96	0.702	72.7	3,928	4	81
69	Sao Tome and Principe	SSA	144	0.525	64.9	1,864	5	79
70	Senegal	SSA	154	0.47	59.6	1,653	471	73
71	Sierra Leone	SSA	177	0.359	48.1	881	227	50
72	Solomon Islands	EAP	143	0.53	68.2	2,172	17	85
73	Somalia	SSA		..	51.5	150	416	9
74	South Sudan	SSA		..	..	..		
75	Sri Lanka	SOA	92	0.715	75.1	5,170	373	98
76	Sudan	SSA	171	0.414	61.8	1,848	1,447	21
77	Swaziland	SSA	141	0.536	48.9	5,104	35	80
78	Syrian Arab Republic	MEN	116	0.648	76	4,674	466	78
79	Tajikistan	ECA	125	0.622	67.8	2,119	194	88
80	Tanzania, United Republic of	SSA	152	0.476	58.9	1,383	1,913	50
81	Timor-Leste	EAP	134	0.576	62.9	5,446	44	22
82	Togo	SSA	158	0.459	57.5	928	195	67
83	Tonga	EAP	95	0.71	72.5	4,153	3	98
84	Tuvalu	EAP		..	67.5	5,650		93
85	Uganda	SSA	161	0.456	54.5	1,168	1,545	57
86	Uzbekistan	ECA	114	0.654	68.6	3,201	589	97
87	Vanuatu	EAP	124	0.626	71.3	3,960	7	80
88	Vietnam	EAP	127	0.617	75.4	2,970	1,458	92
89	Yemen	MEN	160	0.458	65.9	1,820	940	24
90	Zambia	SSA	163	0.448	49.4	1,358	622	48
91	Zimbabwe	SSA	172	0.397	52.7	424	377	65

HDI: Human Development Index; GNI: Gross National Income; World Regions: East Asia & Pacific (EAP), Europe & Central Asia (ECA), Latin America & Caribbean (LAC), Middle East & North Africa (MEN), South of Asia (SOA) and Sub-Saharan Africa (SSA).
Notes: The human development index (HDI), is published by the United Nations Development Program (UNDP). It is a robust composite measure of the average achievement in three basic dimensions of human development namely: a long and healthy life (health), knowledge (education) and a decent standard of living (income). By world regions, 42 (46%) countries are from Sub-Saharan Africa, 18 (20%) from East Asia & Pacific, 10 (11%) from Latin America & Caribbean, 8 (9%) from Middle East & North Africa, 7 (8%) from South Asia and 6 (6%) from Europe & Central Asia. About a third (33 countries) have at least half a million live-births annually, account for over half (58%) of global births and have a median institutional delivery rate of 47% (IQR:35 -65%).
