# Supplementary material for: Risk Factors for Severe Neonatal Hyperbilirubinemia in Low and Middle-Income Countries: A Systematic Review and Meta-Analysis
Source: PLoS One. 2015 Feb 12;10(2):e0117229. doi: 10.1371/journal.pone.0117229 (PMC4326461; doi:10.1371/journal.pone.0117229)
Supplement: S2 Table — (DOCX) [file pone.0117229.s014.docx]

**Table S2. Quality assessment checklist for selected studies***

1. Was the sampling method representative of the target population?

| A. | Non-probability sampling (including: purposive, quota , convenience and snowball sampling) | 0 |
| --- | --- | --- |
| B. | Probability sampling (including: systematic recruitment, simple random) | 1 |

1. Was sample size statistically determined and/or adequately powered?

| A. | No | 0 |
| --- | --- | --- |
| B. | Yes | 1 |

1. Was eligibility criterion clearly defined?

| A. | No | 0 |
| --- | --- | --- |
| B. | Yes | 1 |

1. Was the diagnosis of hyperbilirubinemia objective?

| A. | By history, parental report or visual evaluation solely | 0 |
| --- | --- | --- |
| B. | By bilirubin assay | 1 |

1. Were outcomes measures (Severe jaundice, ABE or kernicterus) clearly defined and assessed?

| A. | No | 0 |
| --- | --- | --- |
| B. | Yes | 1 |

1. Did the statistical methods control for confounding factors (e.g. stratification/ matching adjustment)

when analyzing the associations?

| A. | No | 0 |
| --- | --- | --- |
| B. | Yes | 1 |

Scoring method:

| Grading | 5 or 6 out of 6 | 3 or 4 out of 6 | 0,1 or 2 out of 6 |
| --- | --- | --- | --- |
| Risk of bias | Low | Medium | High |
| Study quality | Good | Satisfactory | Poor |

*Adapted from: Wong WC, Cheung CS, Hart GJ. Development of a quality assessment tool for systematic reviews of observational studies (QATSO) of HIV prevalence in men having sex with men and associated risk behaviours.

*Emerg Themes Epidemiol* 2008;5:23.
