# Supplementary material for: Risk Factors for Severe Neonatal Hyperbilirubinemia in Low and Middle-Income Countries: A Systematic Review and Meta-Analysis
Source: PLoS One. 2015 Feb 12;10(2):e0117229. doi: 10.1371/journal.pone.0117229 (PMC4326461; doi:10.1371/journal.pone.0117229)
Supplement: S3 Table — (DOCX) [file pone.0117229.s015.docx]

**Table S3. Methodological quality of included studies**

|  | First author,  Publication year [ref] | Sampling method | Sample size | Eligibility criterion | NNJ diagnosis | Primary outcome assessment | Adjustment for confounders | Total score | Risk of bias |
| --- | --- | --- | --- | --- | --- | --- | --- | --- | --- |
| 1 | Sodeinde, 1995 [21] | 1 | 0 | 1 | 1 | 0 | 1 | 4 | Medium |
| 2 | Arif, 1999 [22] | 1 | 1 | 1 | 1 | 0 | 1 | 5 | Low |
| 3 | Murki, 2001 [23] | 1 | 0 | 1 | 1 | 1 | 1 | 5 | Low |
| 4 | Agrawal, 2009 [24] | 1 | 0 | 1 | 1 | 1 | 1 | 5 | Low |
| 5 | Kalakheti, 2009 [25] | 1 | 0 | 1 | 1 | 1 | 1 | 5 | Low |
| 6 | Olusanya, 2009 [26] | 1 | 1 | 1 | 0 | 0 | 1 | 4 | Medium |
| 7 | Adebami, 2011 [27] | 1 | 1 | 1 | 1 | 1 | 1 | 6 | Low |
| 8 | Gamaledin, 2011 [28] | 1 | 0 | 1 | 1 | 1 | 0 | 4 | Medium |
| 9 | Ogunlesi, 2011 [29] | 1 | 0 | 1 | 1 | 1 | 1 | 5 | Low |
| 10 | Chawla, 2012 [30] | 1 | 1 | 1 | 1 | 1 | 1 | 6 | Low |
| 11 | Kaur, 2012 [31] | 1 | 1 | 1 | 1 | 1 | 1 | 6 | Low |
| 12 | Scrafford, 2013 [32] | 1 | 1 | 1 | 0 | 0 | 1 | 4 | Medium |
| 13 | Tiwari, 2014 [33] | 0 | 1 | 1 | 1 | 1 | 1 | 5 | Low |
